# Climate change and infectious diseases

**DOI:** 10.1186/s40985-016-0035-2

**Published:** 2016-10-27

**Authors:** Antoine Flahault, Rafael Ruiz de Castaneda, Isabelle Bolon

**Affiliations:** 1 Centre Virchow-Villermé, Descartes School of Medicine, Université Sorbonne Paris Cité, Paris, France; 2 Institute of Global Health, Faculty of Medicine, University of Geneva, Geneva, Switzerland

**Keywords:** Climate change, Infectious diseases, Global health, El Niño/La Niña, Emerging infectious diseases

## Abstract

Global changes are major determinants for infectious diseases, although attributable, part of climate change remains debatable. Vector-borne diseases are prone to be impacted by global warming, although other factors may play a substantial role, evidenced by the dramatic decrease in malaria in the last decades in places where climate change has deep and significant effects. There is now evidence that in some areas of the world, e.g. Horn of Africa, warm El Niño Southern Oscillations (ENSO), which are observed in the South Pacific Ocean, are associated with higher risk of emergence of Rift Valley fever, cholera and malaria and during cold La Niña events, dengue fever, chikungunya and yellow fever. This has been observed for these and other diseases in other parts of the world. For example, seasonal influenza outbreaks have been more intense (i.e. higher number) and more severe (i.e. higher mortality) when concomitant with La Niña events. Since climate scientists have recently observed that climate change is tied to more frequent and more intense ENSO events, we may foresee increases in frequency and severity in emerging infectious diseases in the world.

## Background

Links between climate change and communicable diseases are complex. Climate change is amongst many other determinants, such as environmental, social and political factors that act on transmissibility of diseases. One example, which illustrates this complexity is malaria, for which the number of cases has dramatically decreased during the past decades [1]. This decrease was observed due to large financial investments in the fight to eliminate malaria, although climate change undoubtedly hinders the progress towards elimination. Climate in the future might become more suitable for malaria transmission in the tropical highland regions, as modelled by Caminade et al. [2].

## Main text

We see how El Niño Southern Oscillations (ENSO) in the Southern Pacific may affect the climate in many parts of the world and, as a consequence, also affect communicable diseases. Recent papers [3, 4] have highlighted the risk that climate change may have an influence in increasing intensity and frequency of the El Niño/La Niña phenomenon. The Pacific Ocean is the largest mass of water in the world, so any variations in its temperature have a repercussion on climate in many points of the planet. For example, El Niño has been associated with heavy rainfalls in the horn of Africa over several years and with anomalies in vegetation (wetter than usual) observed from satellites. Linthicum et al. have shown a strong correlation between the El Niño effects and outbreaks of Rift Valley fever (RVF) in the Horn of Africa [5]. RVF is a very severe, arboviral, mosquito-transmitted disease affecting both cattle and humans. In the Horn of Africa, excessive humidity observed from remote sensing, alongside the El Niño phenomenon, is linked to higher probabilities of RVF epidemics.

At the end of 2015, we experienced a strong El Niño phenomenon. If these events happen more frequently and intensely due to climate change, there is a risk of a greater number of outbreaks of emerging or re-emerging infectious diseases. In 2007, El Niño has been found associated with an increase in probability of RVF, cholera and malaria [6] in the Horn of Africa. In other parts of the world such as Bangladesh, temperature rises in the waters of the Gulf of Bengal are tied to re-emerging cholera; for similar reasons, the risk of cholera in Peru has also been increasing. Reversely, El Niño causes drought and heat waves in North-East Brazil and Southeast Asia increasing risks of dengue fever [6].

La Niña is the reverse climatic phenomenon to El Niño. Surprisingly, since it is known as a “cold oscillation”, La Niña will probably also increase in intensity and frequency as a result of climate change [3]. This climatic oscillation is also associated with the emergence of epidemics that have been reported in the recent past.

In May 2004, heatwave and droughts were observed in the coastal areas of Kenya, towards Lamu and Mombasa, two large coastal cities. That period was also the beginning of a large outbreak of chikungunya in these two cities (with reported attack rates of 75 %) prior to its spread to the Indian Ocean [7]. Entomologists have explained how and why droughts can be associated with increases in *Aedes*-borne diseases, such as chikungunya, as well as dengue, Zika and yellow fever [8]. During droughts, due to water scarcity, people are prone to store larger amount of water, outside, in the shadow of their house, for longer time periods, providing shelters to mosquitos’ eggs and larvae.

Vector-bone diseases are not the only maladies linked to El Niño and/or La Niña. Recent studies show that twentieth century pandemics, such as influenza pandemics, were associated with the La Niña phenomenon [9]. This link has been previously studied through seasonal influenza epidemics, and a statistical correlation was found with La Niña, both in the USA and Europe [10]. There is indeed a strong synchronization of seasonal influenza epidemics between Europe and the USA as data collected over several decades demonstrates less than half a week in time difference in epidemic peaks between France and the USA [11]. In addition, there is a correlation between the size of the seasonal influenza epidemics between France and the USA. This intercontinental synchronism points to the possibility that a climatic force could be a factor. Indeed, a positive correlation was found between the size and severity (in terms of mortality) of influenza epidemics in Europe and the USA and La Niña cold oscillations in the South Pacific Ocean [11].

## Conclusions

In conclusion, for over 50 years [12], there has been an increase in outbreaks of emerging infectious diseases, and climate change is probably one of the key drivers of this increase. Vector-borne diseases are amongst those enduring the greatest impact by climate conditions and global warming but airborne transmitted diseases may also be affected. It appears, however, that climate change is not the single determinant for emerging communicable diseases.

## Abbreviations

ENSO: El Niño Southern Oscillations
RVF: Rift Valley fever
